# Characterization of sheep pox virus vaccine for cattle against lumpy skin disease virus

**DOI:** 10.1016/j.antiviral.2014.06.009

**Published:** 2014-09

**Authors:** Eeva S.M. Tuppurainen, Caroline R. Pearson, Katarzyna Bachanek-Bankowska, Nick J. Knowles, Shadi Amareen, Lorraine Frost, Mark R. Henstock, Charles E. Lamien, Adama Diallo, Peter P.C. Mertens

**Affiliations:** a The Pirbright Institute, Ash Road, Pirbright, Surrey GU24 0NF, UK; b Jordan Bio-Industries Centre (JOVAC), PO Box 43, Amman 11941, Jordan; c Animal Production and Health Laboratory, Joint FAO/IAEA Division of Nuclear Techniques in Food and Agriculture, Department of Nuclear Sciences and Applications, International Atomic Energy Agency, Wagramer Strasse 5, P.O. Box 100, A-1400 Vienna, Austria

**Keywords:** Kenyan sheep and goat pox virus, Lumpy skin disease, Vaccine

## Abstract

Lumpy skin disease is of significant economic impact for the cattle industry in Africa. The disease is currently spreading aggressively in the Near East, posing a threat of incursion to Europe and Asia. Due to cross-protection within the *Capripoxvirus* genus, sheep pox virus (SPPV) vaccines have been widely used for cattle against lumpy skin disease virus (LSDV). In the Middle East and the Horn of Africa these vaccines have been associated with incomplete protection and adverse reactions in cattle post-vaccination. The present study confirms that the real identity of the commonly used Kenyan sheep and goat pox vaccine virus (KSGP) O-240 is not SPPV but is actually LSDV. The low level attenuation of this virus is likely to be not sufficient for safe use in cattle, causing clinical disease in vaccinated animals. In addition, Isiolo and Kedong goat pox strains, capable of infecting sheep, goats and cattle are identified for potential use as broad-spectrum vaccine candidates against all capripox diseases.

## Highlights

• KSGP O-240 strain was identified as lumpy skin disease virus.
• Commercially available KSGP O-240 vaccines should be re-characterized.
• The safety of these vaccines in cattle against LSDV should be re-evaluated.
• Two GTPV candidates were identified for use as a broad-spectrum capripox vaccine.

## 1 Introduction

Lumpy skin disease virus (LSDV), sheep pox virus (SPPV) and goat pox virus (GTPV) comprise the *Capripoxvirus* genus within the *Poxviridae* family (Buller et al., 2005). Sheep pox (SPP) and goat pox (GTP) are endemic in northern and central Africa and in large parts of Asia. Lumpy skin disease (LSD) occurs across Africa and has recently been aggressively spreading in the Middle East, despite excessive vaccination campaigns carried out in the region. The latest outbreaks of LSD were reported to the World Organization for Animal Health (OIE) Wahid database from Turkey and Iraq, raising concerns that the disease will continue to spread to Europe and Asia. All cattle breeds, ages and sexes are affected, although the disease is more severe in young animals and cows in the peak of lactation (Weiss, 1968), causing severe production losses throughout the cattle industry.

It is widely agreed that vaccination is the only effective way to control the spread of LSDV in endemic countries. In previously disease-free countries, slaughter of infected and in-contact animals and movement restrictions have been effective, as long as the disease is detected at a very early stage and control measures are implemented without delay. However, if the disease has accidentally gone unnoticed, allowing time for vectors to become infected, it is difficult if not impossible, to eradicate the disease without vaccination. In resource-limited countries, slaughter of infected and in-contact animals is seen as a waste of a valuable source of food and is not usually feasible. In addition, in affected regions, it is often impossible to effectively implement movement restrictions for small and large ruminants (Kitching, 1986). Cross-immunity is known to occur between the members of the genus *Capripoxvirus* (Kitching, 1983). Because SPP and GTP do not occur in southern Africa, only attenuated LSDV vaccines are used against LSDV in the region. Whereas, in central and northern Africa and in the Middle East, where the distribution of SPP, GTP and LSD overlap, attenuated SPPV vaccines, such as KSGP O-240, Yugoslavian RM65 and Romanian SPPV strains, have been used against LSDV (Brenner et al., 2009, Davies, 1991, Kitching, 1986, Somasundaram, 2011).

Because the strain KSGP O-240 infected sheep and goats, causing only mild clinical disease, it was long considered as an ideal vaccine candidate against both SPP and GTP. In addition, it was surprisingly easily attenuated, after only 6 passages on cell cultures (Kitching et al., 1987). Incomplete protection against LSD has been reported in cattle vaccinated with all SPP vaccines (Ali et al., 1990, Ayelet et al., 2013, Brenner et al., 2009, Khalafalla et al., 1993, Somasundaram, 2011). On the other hand, the KSGP O-180 strain, collected from sheep during the same epizootics but at different time points than the KSGP O-240 strain (Davies, 1976, Davies and Otema, 1978), was successfully used in Kenya as a vaccine against SPPV, GTPV and LSDV without adverse reactions. The difference was that KSGP O-180 isolate had been attenuated by passaging the virus 18 times on bovine fetal muscle cells. The efficacy of the vaccine for sheep, goats and cattle was demonstrated by a challenge experiment and in the field (Davies and Mbugwa, 1985).

Lumpy skin disease was reported in Kenya for the first time in 1957 (MacOwan, 1959). The disease was introduced to a mixed cattle and sheep farm near Nakuru by indigenous sheep infected with SPPV originating from the nearby Baringo district of Kenya. Sheep and Ayrshire calves were penned together at night. Soon after arrival, the lambs started to show clinical signs of SPP followed by a similar condition in the calves (Burdin and Prydie, 1959). During the same time period, SPPV was isolated from SPP samples from the Isiolo district and the Kedong Valley (Capstick, 1959). The Isiolo strain is known to experimentally infect cattle (Capstick, 1959) and the Kedong strain has been used as a vaccine for cattle against LSDV in Kenya (Coakley and Capstick, 1961).

In general, capripoxviruses (CaPV) are considered to be very host-specific (Babiuk et al., 2009). In addition to the isolate KSGP O-240, only a few other SPPV and GTPV strains have been known to affect both sheep and goats (Asagba and Nawathe, 1980, Yan et al., 2012). However, no reports exist on CaPV infecting all three species: sheep, goats and cattle. The major difference between the African and the Middle Eastern and Indian SPP and GTP strains seems to be the wider host range of the African isolates (Davies, 1976). The Kenya sheep-1 (KS-1) strain is derived from the attenuated KSGP O-240 vaccine strain (Chand et al., 1994, Gershon and Black, 1989). Recent molecular studies have reported a close relationship between the KS-1 and LSDV, suggesting that KS-1 is actually LSDV (Tulman et al., 2002). Later this finding was confirmed by sequencing the host-specific G-protein-coupled chemokine receptor (GPCR), or RNA polymerase (RPO30) genes, which revealed the phylogenetic grouping of CaPVs (Lamien et al., 2011a, Lamien et al., 2011b, Le Goff et al., 2005, Le Goff et al., 2009). Members of the *Capripox* genus cannot be distinguished using serological methods (Kitching, 2003). A recently published real-time PCR assay provides a simple tool for differentiation of CaPV strains (Lamien et al., 2011b). Here we report the molecular characterization of the virulent Kenyan KSGP O-240 field strain, Isiolo and Kedong SPP isolates and the attenuated KS-1 and KSGP O-240 vaccine strains held in The Pirbright Institute reference virus collection. Selected commercially available SPPV vaccines against LSDV, used for cattle in the Middle East and northern and central Africa, were also analyzed.

## 2 Materials and methods

### 2.1 Virus isolates and vaccines

Virulent KSGP O-240 field strain of 3rd passage (p), Isiolo SPPV (9th p) and Kedong SPPV (2nd p) and the following attenuated vaccine viruses: KS-1 isolate, Kenyavac (KSGP O-240) and Jovivac (RM65) vaccines by Jordan Bio-Industries Centre (JOVAC); and Sheep Pox Vaccine (Romanian SPPV) by Saudi Arabian Veterinary Vaccine Institute (SAVVI), were included in this study (Table 1).

**Table 1:** The origin, host and reference of the virulent and attenuated capripoxvirus strains.

Strain	Origin	Host	References
KSGP 0-240 field strain	Kenya	Sheep	Davies (1976)
Isiolo SPPV	Kenya	Sheep	Capstick (1959)
Kedong SPPV	Kenya	Sheep	Coakley and Capstick (1961)
KS-1 36 3/95	KSGP 0-240	Sheep	Gershon and Black (1989)
Kenyavac	KSGP 0-240	Vaccine	JOVAC^a^
Jovivac	RM65	Vaccine	JOVAC
Sheep Pox Vaccine	Romanian SPPV	Vaccine	SAVVI^b^
SPPV control	Mongolian SPPV (2007)	Sheep	Sample ID: POX-VI-02-07
GTPV control	Mongolian GTPV (2008)	Goat	Sample ID: POX-VI-08-08
LSDV Neethling strain	Dr. Erasmus, ARC-OVI, Onderstepoort, SA	Cattle	Kitching et al., 1989

a JOVAC Jordan Bio-industries Centre.

b SAVVI Saudi Arabian Veterinary and Vaccine Institute.

### 2.2 General capripoxvirus real-time PCR

DNA was extracted from virus suspensions using DNeasy Blood & Tissue Kit (Qiagen, UK) following the manufacturer’s instructions. The presence of viral DNA in the samples was quantified using a previously described general CaPV real-time PCR. Primers and a probe were used in combination with a QuantiFast Probe PCR Kit (Qiagen, Crawley, UK) in a Mx3005p Multiplex Quantitative PCR System (Strategene, Netherlands) (Bowden et al., 2008, Stubbs et al., 2012).

### 2.3 Species-specific real-time PCR

In order to identify which of the three CaPV strains were present in each sample, a species-specific real-time PCR method was used (Lamien et al., 2011b). The PCR assay detects differences in the melting point temperatures for SPPV, GTPV and LSDV, obtained after fluorescence melting curve analysis. It targets a 200 bp region within the GPCR gene. Samples were run on the Mx3005p Multiplex Quantitative PCR System (Strategene, Netherlands) and melting curves were analyzed to determine the CaPV strain.

Mongolian GTPV (2008) was used as a positive control for GTPV, Mongolian SPPV (2007) for SPPV and South African LSDV Neethling strain for LSDV (Kitching et al., 1989). RNase free water was used as a negative control in all PCR runs.

### 2.4 Sequencing

Full length GPCR and RPO30 genes were generated by amplification of overlapping fragments using primers pairs described by Gelaye et al. (submitted in 2014). In each reaction, 4 μl of the viral DNA was mixed with 12.5 μl KOD Hot Start Master Mix (Merck, Germany) and 1 μl (10 μM) of each forward and reverse primer in total volume of 25 μl. The DNA was initially denaturated at 95 °C for 2 min and amplification was carried out in 35 cycles of 95 °C for 20 s, 65 °C for 10 s, 70 °C for 20 s. The amplification products were visualized and assessed for size by agarose gel electrophoresis. All PCR products were purified by GFX™ PCR DNA and Band Purification Kit (GE Health Care, UK). The amplicons were sequenced using the BigDye Terminator v3.1 Cycle Sequencing kit (Applied Biosystems, UK) in a 3730 DNA Analyzer (ABI, UK) according to the manufacturer’s instructions using the same primer sets as for PCR amplification. The resulting sequences were assembled with the SeqMan Pro™ program (Lasergene v.11; DNAStar Inc., USA) and aligned with each other using the CLUSTAL W algorithm (Thompson et al., 1994) in BioEdit 7.0.5.3 (Hall, 1999). Molecular phylogenetic analyses were performed using MEGA 5.2 (Tamura et al., 2011). The evolutionary history was inferred using the Neighbor-Joining method (Saitou and Nei, 1987) and confidence on branching was assessed using bootstrap resampling (1000 replicates) (Felsenstein, 1985). The evolutionary distances were computed using the Kimura 2-parameter method (Kimura, 1980).

## 3 Results

### 3.1 Virulent and attenuated virus isolates

**Fig. 1:**
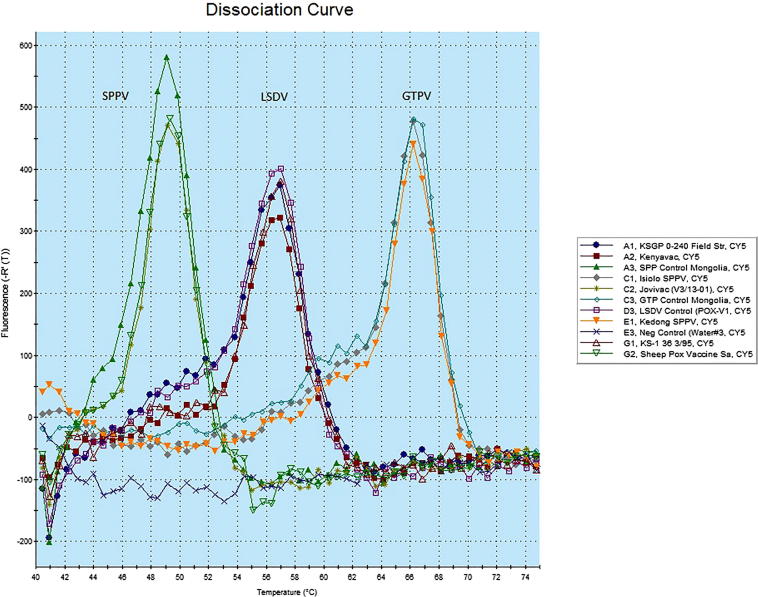
Differences in the melting point temperatures for the virulent and attenuated capripoxvirus strains obtained after fluorescence melting curve analysis, using species-specific PCR method (Lamien et al., 2011b).

Using the species-specific CaPV real-time PCR method, the virulent KSGP O-240 isolate was characterized as LSDV. Isiolo and Kedong SPPV isolates were identified as GTPVs (Fig. 1).

### 3.2 Vaccines

The attenuated KSGP O-240 vaccine virus present in the Kenyavac and KS-1 isolates were identified as LSDVs. The RM65 strain in Jovivac and the Romanian SPPV strain in the Saudi Arabian Sheep Pox Vaccine were confirmed as SPPVs (Fig. 1).

### 3.3 Sequencing data

The sequences of RPO30 and GPCR genes were determined for the six capripoxviruses under study and submitted to GenBank (accession numbers KJ818279 to KJ818292). These were compared with the sequences of capripoxviruses already available on the public sequence databases.

**Fig. 2:**
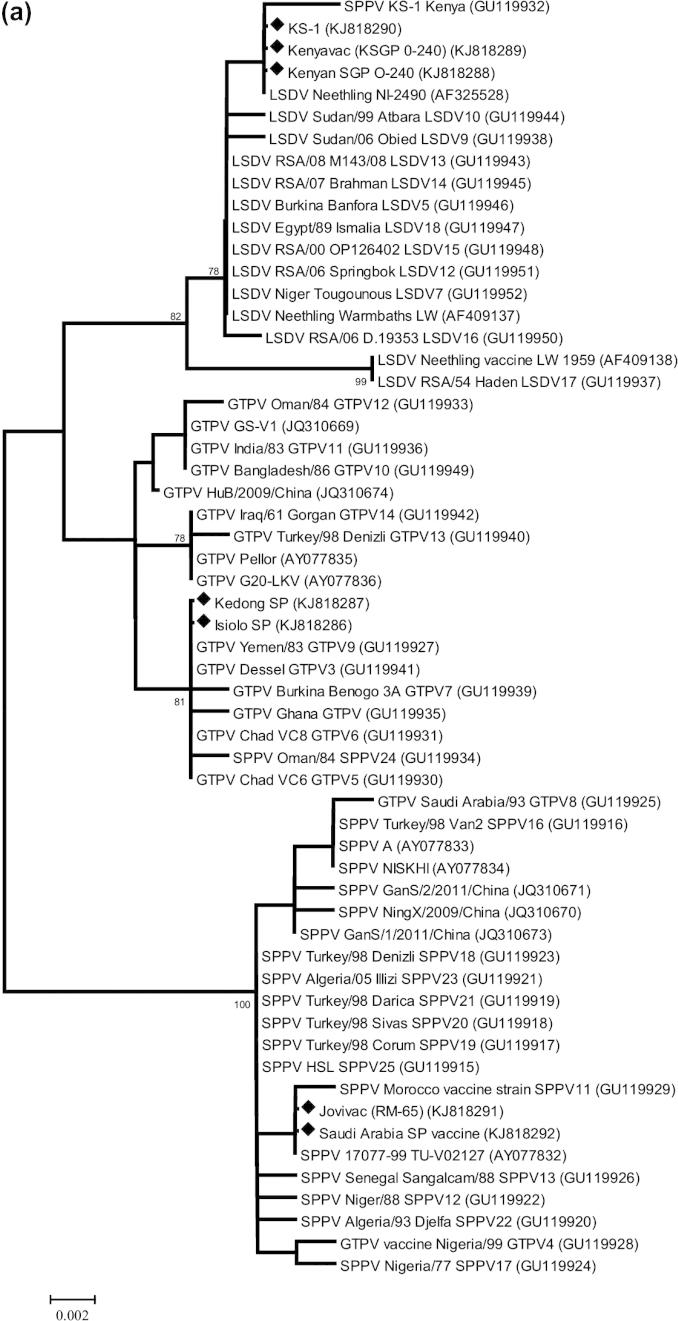

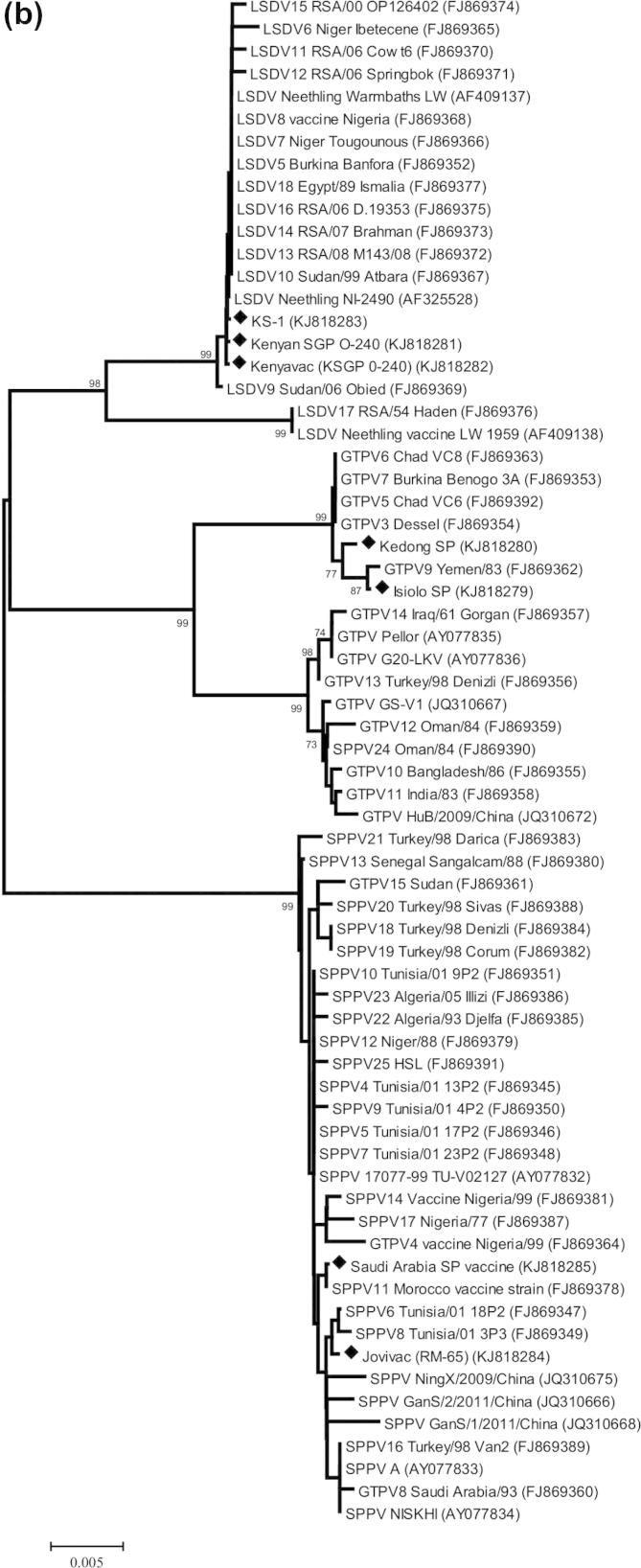
Molecular phylogenetic analysis of (a) the capripoxvirus RNA polymerase subunit (RPO30) gene and (b) the capripoxvirus G-protein-coupled chemokine receptor (GPCR) gene. The percentage of replicate trees in which the associated taxa clustered together in the bootstrap test (1000 replicates) are shown next to the branches. The tree is drawn to scale, with branch lengths in the same units as those of the evolutionary distances used to infer the phylogenetic tree. The evolutionary distances are in the units of the number of base substitutions per site. The sequences determined in this study are marked with a black diamond.

Molecular phylogenetic analyses were performed on the coding regions of the RPO30 gene (57 sequences, 609 positions; Fig. 2a) and the GPCR gene (67 sequences, 1146 positions; Fig. 2b). These results confirmed the identifications made using the real-time PCR. Additionally, the sequence of KS-1 RPO30 gene revealed two A to G nucleotide substitutions between the KS-1 Pirbright isolate and the published sequence (GU119932). These are clearly both A in the new sequence (nucleotide positions 444 and 516 within the RPO30 coding sequence) but do not result in any amino acid substitutions.

## 4 Discussion

The KSGP O-240 strain has long been considered as the SPPV reference virus for comparison with LSDV (Davies, 1982). This strain was chosen for use in vaccines by many vaccine producers because it was one of the CaPV strains listed as a possible seed virus for LSD vaccine in the LSDV chapter of the OIE Manual of Diagnostic Tests and Vaccines for Terrestrial Animals.

Tulman et al. (2002) were the first to report similarities in the pattern of open reading frames of the KS-1 virus and LSDV: ORF 002, 155 and 013 were intact in both KS-1 and LSDV strains, while these regions were disrupted in other SPPVs. In general, the importance of this finding has not been fully appreciated because the origin of the KS-1 strain was not widely recognized to be an attenuated strain of KSGP O-240 strain and therefore LSDV. Because the whole genome of the KSGP O-240 has not yet been sequenced or published, the final confirmation of relationships between KSGP O-240 and LSDV is still to be investigated.

The findings of our study are in agreement with previously reported results: the virulent KSGP O-240, the attenuated KSGP O-240 strain (Kenyavac) as well as the KS-1 isolate were identified as LSDV. The real identity of the vaccine virus explains the easy attenuation of the virus for safe use in sheep and goat vaccines. It is however clear that the level of attenuation of the virus was insufficient for the use of KSGP O-240 for cattle, in which clinical disease was observed post-vaccination (Ayelet et al., 2013, Somasundaram, 2011, Tuppurainen, 2006).

The level of attenuation in Kenyavac is 13–27 passages on lamb testis cells. In a similar KSGP O-240 vaccine, “Tissue Culture Sheep Pox Vaccine” (Veterinary Serum and Vaccine Research Institute, Egypt), that was used against LSDV during the LSD outbreak in 2005–2006 in Egypt, was attenuated three times on choroid plexus cells, followed by three times on lamb fetal lung cells and three times on Vero cells (Tuppurainen, 2006). The level of attenuation is considerably lower than reported for safe use of LSDV in cattle. The LSD Neethling strain required 60 passages on lamb kidney cells and 20 on chorioallantoic membrane (Kitching, 2003). The LSD Madagascan strain was passaged 101 times in rabbit kidney and 5 times in fetal calf kidney cells (Kitching, 2003).

After experimental infection with LSDV only half of the infected cattle developed clinical disease (Annandale et al., 2013, Osuagwuh et al., 2007, Tuppurainen et al., 2005) and silent infections without skin lesions are known to commonly occur in field outbreaks of LSDV (Davies, 1976). In above mentioned animal experiments, a minimum number of six, highly susceptible, naïve animals were required in order to produce clinical disease in cattle challenged with LSDV via an intravenous and/or intradermal route. This gives guidelines on the animal numbers required for safety and efficacy experiments for CaPV vaccines.

It was believed that due to the cross-protection within the genus, any CaPV isolate could be used as a vaccine against LSDV. However, experience in the field setting indicates the superiority of LSDV vaccines when compared to SPPV vaccines against LSDV. In addition, according to the previous recommendations for SPPV vaccines for cattle against LSDV, the suggested titre for RM65 or Romanian SPPV vaccines is 10–50 times the recommended dose for sheep (10^2.5^ TCID_50_), whereas for KSGP O-240 strain an immunizing dose of 10^3.5^ TCID_50_ was considered desirable for field vaccination campaigns (Davies, 1991). However, these recommendations may be out-of-date and the efficacy of the vaccine should be re-tested by a challenge experiment in a controlled environment, using sufficiently sensitive testing methods such as real-time PCR and a sufficient number of fully susceptible cattle.

Due to difficulties controlling LSDV by vaccination in the Horn of Africa and the Middle East (Ayelet et al., 2013, Brenner et al., 2009, Tuppurainen, 2006), and taking into consideration the distinct threat of incursion of all CaPV diseases to Europe and Asia, a new generation of effective and safe vaccines against LSDV, SPPV and GTPV are urgently required. Ideally the vaccine should be affordable and available for use both in endemic and non-endemic countries without adverse effect on global trade of live animals and their products. None of the currently available CaPV vaccines provides total protection against LSDV for all vaccinated individuals, which is a clear disadvantage for control of a vector-borne disease.

In the OIE’s Manual Chapter 2.4.14 – Lumpy skin disease, the KSGP O-240 strain is mentioned as one of the four CaPV vaccine strains used for cattle against LSDV. The aim of this report was to confirm and highlight the most recent molecular findings, indicating that the KSGP O-240 vaccine strain is LSDV which at the low level of attenuation is still virulent for cattle. Consequently, the identity of the virus in all of the commercially available KSGP O-240 vaccines is likely to be LSDV instead of SPPV and characterization of the vaccine virus should be carried out before use in cattle. Clinical disease detected in KSGP O-240 vaccinated cattle is more likely to be caused by insufficient attenuation of the vaccine virus than incomplete protection and therefore the safety of the vaccines should be re-evaluated before the vaccine is used for cattle. Additionally, the use of virulent vaccine may lead to the spread of the vaccine virus itself via arthropod vectors. However, sufficiently attenuated KSGP O-240 strain is likely to afford protection for cattle equivalent to other LSDV vaccines.

Due to their broad host-range the Isiolo and Kedong isolates may provide an alternative vaccine candidate that is effective against all capripox diseases. Both isolates were collected from infected sheep, molecular studies identified both as GTPVs. Most phylogenetic studies suggest that GTPV is more closely related to LSDV than SPPV is to LSDV (Lamien et al., 2011a, Le Goff et al., 2009). In addition, the Isiolo strain has been shown to experimentally infect cattle, while the Kedong vaccine strain protects cattle against LSDV. This warrants further investigation of the suitability, efficacy and safety of the Isiolo and Kedong GTP strains, as well as sufficiently attenuated KSGP O-240 and O-180 strains as a basis for affordable broad-spectrum vaccines against LSDV, SPPV and GTPV.
